# Pregnancy in non-palliated functionally single ventricle: challenges of management in resource-poor settings

**DOI:** 10.11604/pamj.2020.35.6.18442

**Published:** 2020-01-09

**Authors:** Desrie Delsol-Gyan, Ernest Aniteye, Samuel Oppong, Ernest Ofosu-Appiah, Frank Edwin

**Affiliations:** 1 School of Medicine, University of Health and Allied Sciences, Ho, Ghana; 2 National Cardiothoracic Centre, Korle Bu Teaching Hospital, Accra, Ghana; 3 School of Medicine and Dentistry, University of Ghana, Accra, Ghana

**Keywords:** Single ventricle, congenital heart disease, pregnancy

## Abstract

Women with complex functionally univentricular hearts rarely survive into adulthood without corrective or palliative surgery. Reports of pregnancy outcome in this group of patients in resource-poor settings are sparse. We report a case of unrepaired pulmonary atresia ventricular septal defect (VSD) with major aorto-pulmonary collateral arteries (MAPCA) who survived into adulthood and was able to complete a successful pregnancy in a resource-poor country.

## Introduction

The term functionally single ventricle refers to a spectrum of congenital heart defects in which the ventricular mass, which may be anatomically single or double, does not lend itself to safe partitioning in a way that commits one ventricular pump to the systemic circulation, and the other to the pulmonary circulation [1]. Thus a patient may possess two ventricles and yet manifest single ventricle physiology if it is not surgically feasible to partition the circulation in a way that commits either ventricle exclusively to the systemic or pulmonary circulation. Unlike the biventricular circulation in which the systemic and pulmonary circulations are connected in series, in a single ventricle physiology, the systemic and pulmonary circulations are connected in parallel, being supplied by the output from the combined ventricular mass. The ventricular ejection is physiologically partitioned between the systemic and pulmonary vascular beds depending on the relative resistances between the two vascular beds. An increase in flow to one vascular bed will only occur at the expense of a reduced flow to the other vascular bed. As a corollary, the oxygen saturation in the aorta and pulmonary arteries are the same. The specific functionally single ventricle lesions include tricuspid atresia, hypoplastic left heart syndrome, double-inlet left ventricle, pulmonary atresia with intact ventricular septum, unbalanced atrioventricular septal defects, and several others.

Uncorrected pulmonary atresia (PA) with ventricular septal defect (VSD) falls in this category as both the systemic and pulmonary blood flows are derived the aorta which receives ejection from the combined mass of right and left ventricles through a large sub-aortic VSD. Pulmonary atresia with VSD and major aorto-pulmonary collateral arteries (MAPCA) is the most extreme form of tetralogy of Fallot (TOF) representing 5% to 10% of TOF patients [2]. The survival rate without surgical repair is reported to be 50% at 1 year of age and 8% at 10 years [3]. Survival into adulthood without palliation is rare; successful pregnancy in such patients is even rarer. Pregnancy is accompanied by dramatic changes in systemic vascular resistance and venous return from the late second trimester onwards. Pregnancy in patients with uncorrected PA VSD MAPCA is fraught with many challenges which are magnified in resource-poor settings. These challenges and their management are not usually encountered in routine clinical practice in developing countries. Current available guidelines are in reference to specialized adult congenital heart disease (ACHD) centers, largely unavailable in resource-poor settings. We report our experience in successfully managing pregnancy in a patient with uncorrected PA VSD MAPCAs in Accra, Ghana.

## Patient and observation

Ghana's National Cardiothoracic Centre was officially inaugurated in April of 1992. In 1983 when the patient was four years of age, she was part of a selected group of children who received philanthropic support to travel to Europe for treatment of cyanotic CHD. She was prepared for surgery with a preoperative diagnosis of truncus arteriosus (common arterial trunk). At surgery, a solitary systemic arterial trunk arising from the ventricular mass was found. The pulmonary blood flow was derived from large collateral vessels arising from the descending aorta. Because the patient's saturation was satisfactory (above 80% most times) no intraoperative intervention was deemed necessary. The patient returned home after an uneventful recovery to be followed up locally. She maintained satisfactory saturations and fairly adequate exercise tolerance throughout childhood. She underwent phlebotomy on three occasions on account of hyperviscosity. Although counselled about the risks of pregnancy, she was insistent on having a child. At age 32 years, she became pregnant but miscarried in the first trimester. One year later, she presented to our clinic with a seven week pregnancy.

At the time of the pregnancy, she was not on any medication and had only mild effort intolerance (NYHA II). The oxygen saturations ranged between 86%-89%. Her blood pressure was 101/69mmHg with a pulse rate of 86 per minute. Her hemoglobin was 18.3g/dl (hematocrit of 54.9%). The transthoracic echocardiography was in keeping with her intraoperative findings in childhood: usual veno-atrial and atrioventricular connections, diminutive outlet portion of the right ventricle without continuity with an arterial segment, both ventricles connected to a 40mm solitary arterial trunk through a 27mm sub-arterial VSD. No intra-pericardial pulmonary artery was found. Large collateral vessels (MAPCAs) arising from the descending aorta appeared to be the source of pulmonary blood flow, with origin stenosis of the left-sided MAPCA. The ventricular function was good with ejection fraction of 76%. There were echocardiographic signs of pulmonary hypertension. The risks of pregnancy to mother and fetus were once again emphasized and termination discussed as an option but the patient preferred to continue with the pregnancy.

A multidisciplinary specialist team consisting of a cardiologist, obstetrician/gynecologist, and cardiac anesthetist as the core team members was formed to draw a management plan for the pregnancy, deliver, and puerperium. The patient, being a house wife, was advised to reduce her chores at home in keeping with her comfort. The patient tolerated the pregnancy relatively well, maintaining oxygen saturations between 84% and 88% until the 28^th^ week. At 28 weeks gestation, her effort tolerance worsened, her oxygen saturation dropped to 80% and the hemoglobin rose to 19.9g/dl. She was hospitalized and placed on bed rest, close surveillance, and regular fetal monitoring. She was given elastic compression stockings and anticoagulated with warfarin (target INR between 2 and 3) to counter the risk of thromboembolism. The initial pelvic scan demonstrated no obvious fetal abnormalities but subsequent scans showed signs of oligohydramnios. The findings suggested slow amniotic fluid loss. The decision was thus taken at 34 weeks to deliver the fetus. An elective Caesarean Section was performed at 34 weeks of gestation under general anesthesia. A female infant, weighing 1.2 kg was delivered with APGAR scores of 6 and 7 (first and fifth minutes).

**Figure 1:**
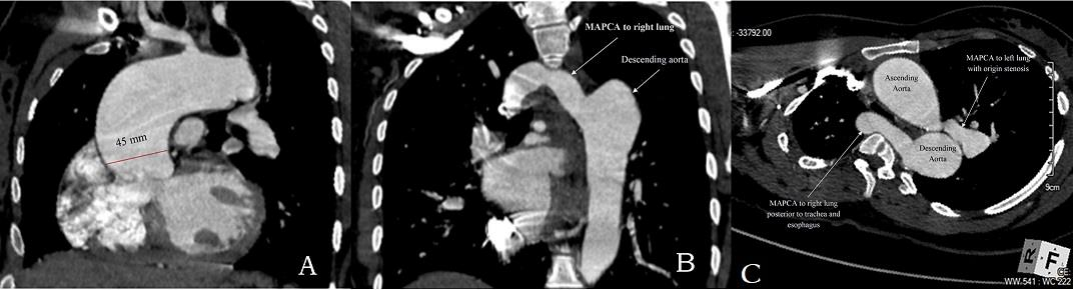
(A) biventricular origin of the dilated ascending aorta (45 mm) with 50% overriding; (B) MAPCA supplying right lung originates from the descending aorta and passing posterior to trachea and esophagus (panel C) to reach the right side; (C) MAPCA to right lung passing posterior to trachea and esophagus. MAPCA to left lung demonstrates severe origin stenosis

On the second postpartum day the patient developed congestive heart failure. She was treated with diuretics (furosemide and spironolactone) with good response and was discharged a week later. The baby was initially nursed in the neonatal intensive care unit. Echocardiography on the baby prior to discharge showed no CHD. Post-delivery evaluation of the mother showed good ventricular function on echocardiogram. A computed tomographic angiography was performed a year after delivery to evaluate the sources of pulmonary blood flow (Figure 1A). The ascending aorta measured 45mm with usual branching pattern. The right lung was supplied by an 18mm MAPCA from the descending aorta (Figure 1B). The left lung was supplied by another MAPCA measuring 20mm with origin stenosis (Figure 1C). After six years post-delivery follow up, both mother and child are doing well. The mother's effort tolerance has diminished moderately but she is still able to care for the child and perform her usual daily chores reasonably well.

## Discussion

Current guidelines recommend that women with CHD should consult an ACHD specialist before becoming pregnant. Also, the management of pregnant patients with cyanotic CHD should be undertaken in an ACHD center by a multidisciplinary team that includes ACHD specialists and maternal-fetal medicine obstetricians with expertise in caring for women with heart disease [4]. In resource-poor settings, these ideals are not attainable due to infrastructure and manpower constraints. Management of such patients in these settings is often accomplished by drawing upon the expertise of practitioners across various departments in a tertiary facility who must work together to achieve optimal outcomes for the patient. During childhood, this patient's diagnosis was truncus arteriosus type IV. The original categorization of truncus arteriosus by Collett and Edwards [5] included a type IV variant characterized by the complete absence of the intra-pericardial pulmonary arteries. Type IV truncus arteriosus is now recognized to be a form of PA VSD [6].

Pregnancy in patients with PA VSD MAPCA carries substantial maternal and fetal risks [7] as well as a risk of CHD in the newborn of 13.4% [8]. Surgical intervention in the form of anatomical repair or palliation that eliminates or significantly ameliorates cyanosis and secondary erythrocytosis has a modifying influence on the pattern of risk observed in pregnant patients with PA VSD MAPCA. In unrepaired patients like ours, maternal mortality and morbidity tends to be low while complications occur mainly in the fetus [7]. A high rate of fetal loss is common as occurred with this patient in the first pregnancy. Maternal cyanosis carries substantial risk to the fetus with a higher chance of fetal loss in the first trimester, prematurity, and small-for-gestational-age neonates. Successful pregnancy is more likely if cyanosis is minimal or absent, and erythrocytosis is moderate or less before pregnancy. Both of these are uncommon findings in the unrepaired patient with PA VSD MAPCA. In one study, the live birth rate was just 8% if the pre-pregnancy hemoglobin exceeded 20g/dl whereas it was 71%, if the hemoglobin was 16g/dl or less, if the maternal oxygen saturation was 85% or less before pregnancy and only 12% of infants were born alive [9]. In this regard, our patient had a borderline fetal risk.

The systemic and pulmonary blood flows in this patient were derived from the solitary arterial trunk with similar oxygen saturations occurring in both the systemic and pulmonary vascular beds. The cardiac ejection by both ventricles into the arterial trunk is partitioned between the systemic and pulmonary vascular beds according to the relative vascular resistance between the systemic and pulmonary vascular bed. The circulation, in essence, is that of a functionally single ventricle. A decrease in systemic vascular resistance (vasodilatation) will increase systemic blood flow at the expense of pulmonary blood flow and peripheral oxygen saturation. As pregnancy advances and systemic vascular resistance falls, hypoxemia may worsen because of increased systemic blood flow at the expense of pulmonary blood flow [4]. We believe this is what happened to the patient when she reached the 28^th^ week of gestation. The worsening hypoxemia and erythrocytosis decreased her effort tolerance and further compromised an already precarious intrauterine growth. Both chronic cyanosis and pregnancy are prothrombotic states. When the patient was admitted for bed rest and close monitoring, it was felt that her thromboembolic risk was heightened and so compression stockings and anticoagulation were deemed necessary. Though anticoagulation is considered useful in pregnant cyanotic patients on bed rest, there is no consensus on this issue [4].

Between the 28^th^ and 34^th^ weeks, there was concern for the mothers worsening hypoxemia, increasing hematocrit and deteriorating effort tolerance. Supplemental oxygen was administered and close fetal monitoring observed. The planned mode and timing of delivery for this patient was vaginal delivery under epidural analgesia at term. This had to be revised when oligohydramnios became evident at 34 weeks gestation. The choice of anesthetic technique is important considering the profound effects anesthetic agents have on systemic vascular resistance and the implications for pulmonary blood flow and oxygen saturation in this patient. The hemodynamic goals during anesthesia were to maintain sinus rhythm, keep the systolic blood pressure above 100mmHg (or mean above 70mmHg) and prevent fluctuations beyond 20% of baseline. Suspected decreases in SVR were treated with norepinephrine by infusion. Although a slowly titrated epidural anesthetic was initially contemplated, issues with prior anticoagulation swung the decision towards general anesthesia. Spinal anesthesia was not favored because of the potential for rapid onset of vasodilatation and consequent increase in right-to-left shunting with worsening cyanosis.

The usual hemodynamic changes occurring after delivery can prove hazardous for patients with functionally single ventricle physiology. Following delivery, extrusion of blood from the contracted uterus and decompression of the inferior vena cava results in a massive increase in venous return. Furthermore, contraction of the utero-placental vascular bed following delivery abruptly increases systemic vascular resistance. These hemodynamic changes may increase the cardiac output by as much as 80% [4] in the normal biventricular circulation connected in series. For the patient with functionally single ventricle physiology, these hemodynamic changes may be poorly tolerated. Ostensibly, our patient's circulation could not cope with the sudden combined increases in venous return and systemic vascular return; she developed heart failure as a result, prolonging her intensive care unit stay by seven days. Our experience emphasizes the point that close attention in the intensive care unit is required to manage the dysfunctional peripartum physiology of patients with functionally single ventricle.

## Conclusion

The course and outcome of this patient with unrepaired VSD PA MAPCA illustrates the importance of teamwork and collaboration among departments in resource-poor settings lacking a dedicated ACHD unit as the preferred care facility. In pregnant patients with functionally single ventricle, clinicians should expect a decreasing systemic vascular resistance favoring enhanced right-to-left shunting and worsening cyanosis in the third trimester. In the immediate post-partum period, a combination of increased systemic vascular resistance and a large increase in venous return may lead to decompensated congestive heart failure. Close surveillance during pregnancy, delivery and the puerperium is essential in ensuring a favorable outcome.

## Competing interests

The authors declare no competing interests.
